# Mechanistic Exploration of *Smilax glabra* Roxb. in Osteoarthritis: Insights from Network Pharmacology, Molecular Docking, and In Vitro Validation

**DOI:** 10.3390/ph17101285

**Published:** 2024-09-27

**Authors:** Sidra Ilyas, Chae Yun Baek, Abdul Manan, Yeojin Choi, Hee-Geun Jo, Donghun Lee

**Affiliations:** 1 Department of Herbal Pharmacology, College of Korean Medicine, Gachon University, 1342 Seongnamdae-ro, Sujeong-gu, Seongnam-si 13120, Republic of Korea; sidrailyas6@gachon.ac.kr (S.I.); cyning20@gachon.ac.kr (C.Y.B.);; 2 Department of Molecular Science and Technology, Ajou University, Suwon 16499, Republic of Korea; 3 Naturalis Inc. 6, Daewangpangyo-ro, Bundang-gu, Seongnam-si 13549, Republic of Korea

**Keywords:** deep learning, network pharmacology, molecular docking, *Smilax glabra* Roxb., arthritis

## Abstract

**Background**: Arthritis, a debilitating joint disease, remains a significant global health burden. This study uncovers the therapeutic potential of the medicinal plant *Smilax glabra* Roxb. (SGR) in attenuating progression of disease by modulating immune responses. **Methods**: Through computational approaches, key bioactive compounds in SGR were identified by using freely available databases: TCMSP, TCMID, HIT2.0, HERB, and INPUT in order to elucidate their underlying mechanisms of action. Therapeutic targets for the disease have been retrieved by TTD, GeneCard, and OMIM databases. The STRING database was used to analyze the protein–protein interactions (PPI) of intersecting genes. Gene ontology (GO) and Kyoto Encyclopedia of Genes and Genomes (KEGG) enrichment analyses were performed to reveal the functional roles of genes. Mcule was used for molecular docking and binding affinity of compounds and targets were evaluated by DeepPurpose model. ALP activity, cell viability assay, TRAP staining were also performed. **Results**: A total of 14 active SGR compounds with 59 common targets for arthritis have been identified. These targets have a major role in controlling biological processes such as wound healing, oxygen responses, and chemical stimuli. Molecular docking by Mcule platform demonstrated that quercetin and β-sitosterol showed higher binding energy affinities with TNF, TP53, PTGS2, and JUN as compared to other targets. To explore the complex relationship between compounds and targets, pre-trained Davis and KIBA models were used to predict the affinity values of selected compounds. In MC3T3-E1 cells, ALP activity was significantly increased and bone marrow macrophages (BMM) showed a low number of TRAP-positive cells in SGR-treated cells. **Conclusions**: Our findings demonstrate that SGR effectively inhibits/regulates inflammatory responses, prevents cartilage degradation, promotes bone regeneration, and can be used as a promising candidate for the development of novel arthritis treatment.

## 1. Introduction

Arthritis, a broader term encompassing diseases that induce inflammation of the synovium, joint pain, cartilage deterioration, swelling, stiffness, and subsequent bone remodeling, is a leading cause of disabilities worldwide [1]. It destroys all joints, including articular cartilage, subchondral bone, ligaments, capsule, and synovial membrane if left untreated and can lead to various defects and disabilities. Osteoarthritis (OA) and rheumatoid arthritis (RA) are the most common types, resulting from joint wear and tear and/or the immune system mistakenly attacking joints. Both have distinct etiologies and pathophysiological mechanisms; however, at the molecular level, they have intriguing overlaps and shared pathways. Despite advancements in management, effective disease-modifying therapies remain elusive. The underlying pathophysiology involves a complex interplay of mechanical stress, inflammatory responses, and extracellular matrix degradation. The traditional approaches for arthritis treatment include a combination of herbs, hyaluronan, corticosteroids, drugs, vitamins, antagonists, paracetamol, and chondroitin sulfate, which may help in relieving pain to improve joint function, but these treatments are ineffective for cartilage repair as they only provide symptomatic relief [2,3,4,5,6]. For severe arthritis cases, a combination of physical and systemic therapies may be required. Given the substantial impact of arthritis on quality of life and healthcare costs, there is an urgent need for novel therapeutic interventions [7].

Arthritis-related inflammation generates pro-inflammatory cytokines such as TNF-α, IL-6, and interleukin (IL)-1β [8]. In addition, ROS produced during the inflammatory process causes damage to tissues and cells. Several natural products have emerged as promising sources of anti-inflammatory, antioxidant, and chondroprotective properties [9]. In this context, *Smilax glabra* Roxb. (SGR), a rhizome of the Liliaceae plant, has garnered attention and is widely used to treat a wide range of diseases, including joint pain and inflammation [10]. Approximately 190 compounds in SGR that comprise organic acids, naphtha, flavonoids, sterols, phenylpropanoids, and polysaccharides have been identified that have pain-relieving, anti-inflammatory, detoxifying, antioxidant, immunosuppressive, and detoxifying roles in attenuating atherosclerosis and preventing lipid peroxidation [11,12,13]. SGR extract may mitigate pathological oxidative stress by decreasing ROS and oxidized glutathione level (GSSG) generation by enhancing the activities of antioxidant enzymes such as superoxide dismutase (SOD) and catalase (CAT) [14]. Astilbin extracted from SGR possesses antibacterial, antioxidative, and anti-inflammatory properties and can be used for the management of autoimmune disorders [15,16,17]. The mechanism of action involves the blocking of the MAPK and NF-κB signaling pathways as both pathways are involved in osteoclastogenesis [15,18]. The compounds extracted from SGR also act as quality markers for standardized use in therapeutic applications [7].

Many countries in Asia have used SGR as a folk medicine for the treatment of liver disorders, coronary heart disease, hyperglycemia, inflammation, and syphilis [10]. However, the underlying mechanism of SGR and its therapeutic potential in arthritis remains largely unexplored. Therefore, this study aims to investigate the anti-arthritis effects of SGR in order to elucidate its underlying molecular mechanism and its therapeutic roles by computational and experimental approaches. We intend to identify the active compounds with potential targets, assessing their interactions by using compounds and target databases that may provide the opportunity for pinpointing promising compounds and targets that could be involved in underlying mechanisms for treating arthritis and inflammation.

## 2. Results

### 2.1. Active Compounds and Targets of SGR

SGR compounds were identified based on the TCMSP (74), TCM-ID (61), HIT (34), HERB (111), and INPUT (60) databases and a total of 340 compounds were retrieved. Compounds that have satisfied certain pharmacological characteristics (OB ≥ 30, DL ≥ 0.18) and ADME properties validated by Swiss Target Prediction and the TCMSP database were selected, narrowing down the candidates to 17 active compounds. The selected SGR compounds are listed in Table 1. A detailed pharmacological and molecular property profiling of selected SGR compounds is presented in Supplementary Table S1.

### 2.2. Potential Targets of Arthritis

A comprehensive search across the OMIM, GeneCards, and TTD databases yielded a total of 5156, 833, 31 disease targets, respectively. The downloaded data were processed for normalization by merging, removing duplicates, and finally 787 potential targets were obtained.

### 2.3. Common Target Determination and Network Construction 

**Figure 1:**
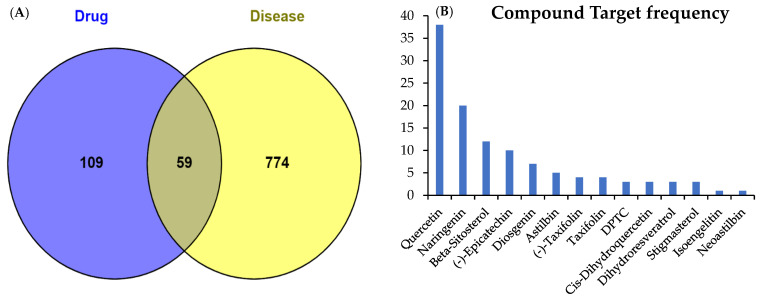
A Venn plot of active compounds of SGR and disease target genes. (**A**) The 59 Overlapping targets identified between two sets. (**B**) The frequency of compounds and disease targets. The compound (2*R*,3*R*)-2-(3,5-dihydroxyphenyl)-3,5,7-trihydroxychroman-4-one was represented as DPTC.

**Figure 2:**
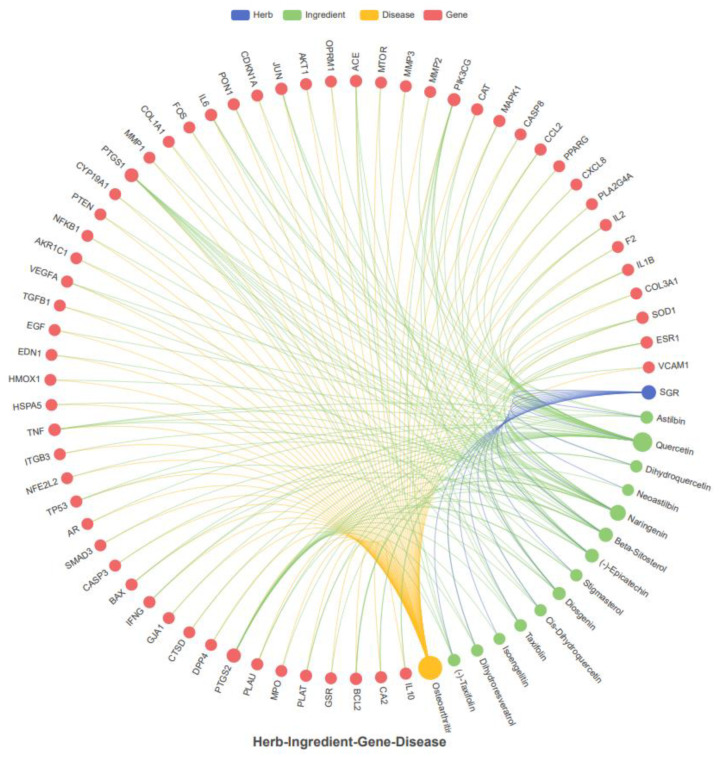
The construction of the SGR-compound-gene-disease network. The active compounds of SGR (green) and disease targets (red) are displayed. The compound (2*R*,3*R*)-2-3,5-dihydroxyphenyl)-3,5,7-trihydroxychroman-4-one was represented as DPTC.

For disease-associated target prediction, a Venn diagram was plotted for the identification of overlapping genes between SGR compounds and arthritis targets (Supplementary Tables S2 and S3). Results showed that 59 targets were shared between SGR and the disease (Figure 1A). The frequency of active compounds and disease targets were shown in Figure 1B. The active compounds with corresponding targets were imported into Cystoscope 3.10.0 software to construct a SGR-compounds-target-disease network. Among them, diosgenin, cis-dihydroquercetin, taxifolin, (2*R*,3*R*)-2-(3,5-dihydroxyphenyl)-3,5,7-trihydroxychroman-4-one, isoengelitin, neoastilbin, astilbin, naringenin, beta-sitosterol, dihydroresveratrol, quercetin, stigmasterol, (-)-epicatechin, and (-)-taxifolin showed higher interaction, whereas sitosterol, enhydrin, and 4,7-dihydroxy-5-methoxyl-6-methyl-8-formyl-flavan were excluded since they lacked common targets in the network (Supplementary Figure S1). The SGR-compound-gene-disease (herb-ingredient-gene-disease HIGD) network was constructed, which showed that predicted targets exhibited synergistic effects and might be involved in arthritis treatment (Figure 2).

### 2.4. Network Visualization and Identification of Hub Targets

**Figure 3:**
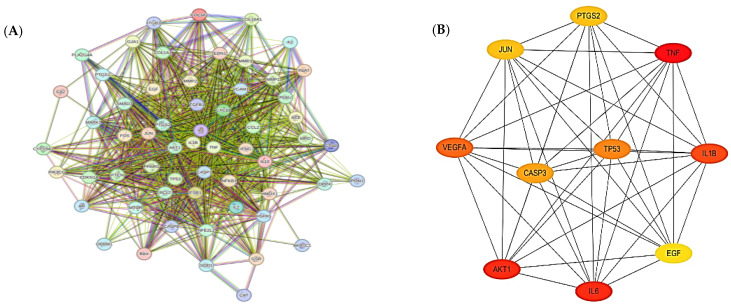
Protein–protein interaction (PPI) network of common targets generated by String database. Colored lines represent known and predicted interactions (light blue: curated databases; dark purple: experimentally determined; green: gene neighborhood; red: gene fusions; dark blue: gene co-occurrence; yellow: text mining; black: co-expression; light purple: protein homology). (**A**) PPI network of 59 common targets. (**B**) Top 10 hub targets ranked by degree (DC) method were predicted by CytoHubba.

Potential therapeutic targets (59) were analyzed by using the STRING database to obtain a protein–protein interaction (PPI) network to explore the relationship between arthritis-related targets. In the PPI relationship network, 85 nodes with 858 edges were obtained (Figure 3A). Biological interactions showing various attributes using the STRING database are shown in Supplementary Table S4. This PPI network was imported into Cytoscape 3.10.0 using the Cytohubba plug-in, and the top 10 core targets, *TNF*, *AKT1*, *IL6*, *IL1B*, *VEGFA*, *TP53*, *CASP3*, *PTGS2*, *JUN*, and *EGF*, were identified as the hub genes/markers that may play a key role for arthritis treatment (Figure 3B).

### 2.5. Functional Annotation of Common Target Genes

**Figure 4:**
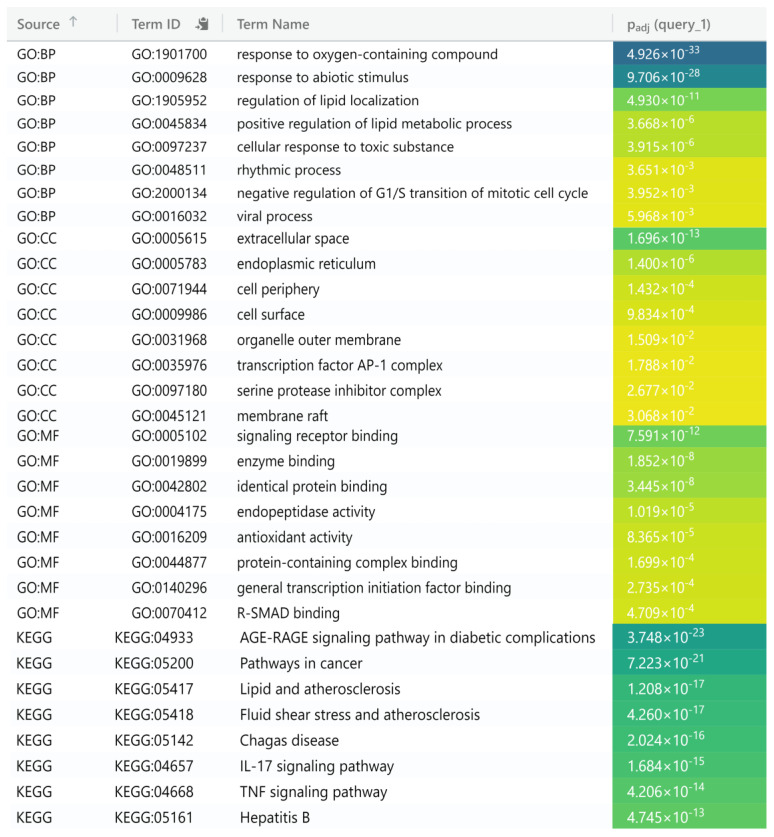
Gene enrichment analyses; GO and KEGG path of potential targets. Top 8 GO significant terms with biological processes (BP), molecular function (MF), cellular component (CC), and first 15 KEGG pathways depicted.

A set of 59 genes submitted to g-Profiler for Gene Ontology (GO) enrichment analysis to obtain information about molecular functions (MFs), cellular components (CCs), and biological processes (BPs) was filtered by considering a *p*-adjusted value < 0.05. A total of 1076 BPs, 35 CCs, 50 MFs, and 112 KEGG entries were identified. The top three BPs featured a cellular response to chemical stimulus, response to oxygen levels, and the regulation of wound healing. The top three MFs consisted of cytokine receptor binding, identical protein binding, and antioxidant activity. The top three CCs comprised the extracellular region, cell periphery, and membrane raft. KEGG-enriched pathways (*p*-adjusted value < 0.05) mainly include the AGE-RAGE signaling pathway, lipids, and atherosclerosis, suggesting numerous targets associated with the progression and onset of osteoarthritis (Figure 4).

### 2.6. Molecular Docking 

**Figure 5:**
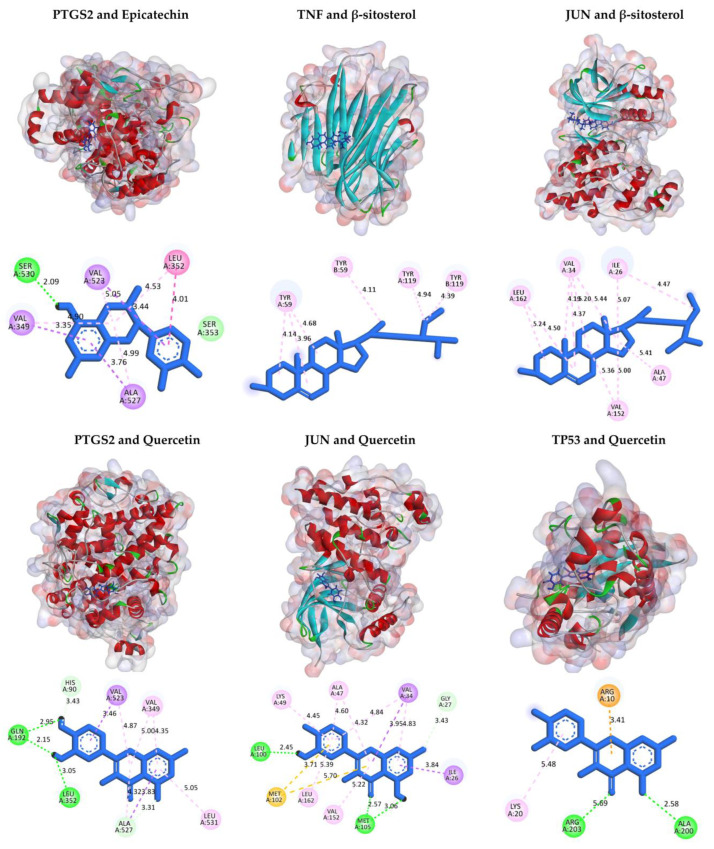
Two-dimensional and three-dimensional interactions of docked complexes of active compounds of SGR and target proteins with their binding residues visualized and predicted by BIOVIA Discovery Studio where van der Waals interactions (bright green), conventional hydrogen bonds (solid green), Pi-sigma (purple), Pi-alkyl (light pink), Pi-sulfur (orange), and Pi-cation (light orange) interactions were represented by spoked lines.

The four active compounds, quercetin, naringenin, beta-sitosterol, and epicatechin, docked against nine hub genes (*TNF*, *AKT1*, *IL6*, *IL-1β*, *VEGFA*, *TP53*, *CASP3*, *PTGS2*, and *JUN*) to determine binding energies (Table 2). Quercetin showed higher binding energy affinities with *TNF*, *TP53*, *PTGS2*, and *JUN* than other target genes. Similarly, β-sitosterol exhibited stronger binding energy values to *TNF* and *JUN* (Figure 5). The higher the binding affinity of active compounds with targets is, the more likely SGR has potential and can be used for the treatment of OA. 

### 2.7. Compounds and Target Binding Affinity Prediction 

To explore the complex relationship between compounds and targets, pre-training Davis and KIBA models were used to predict the affinity values. The KIBA dataset comprised 2111 compounds and 229 proteins with 118,254 binding affinity values, whereas the Davis dataset had 68 active compounds and 442 proteins with 30,056 binding affinity scores. The prediction interaction values of all compounds determined computationally are shown in Supplementary Table S5. Based on the docking binding energies, the compound target binding affinities of selected compounds are shown in Table 3. The length distribution of SMILES and protein sequences are shown in Supplementary Figure S2. 

### 2.8. ALP Activity, Cell Viability, and TRAP Staining 

**Figure 6:**
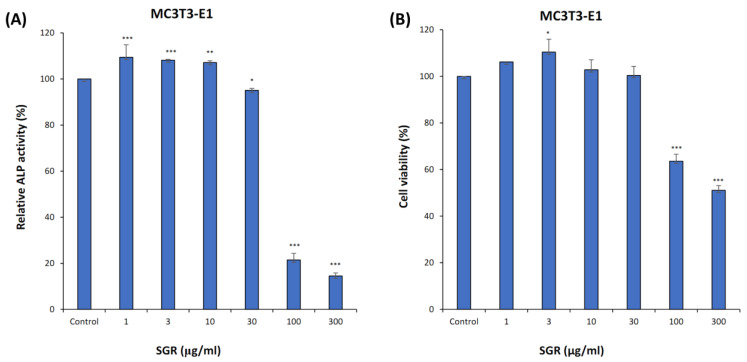
Effects of SGR extract on MC3T3-E1. Cells in osteoblast induction medium were treated with SGR extract (1–300 μg/mL) for 4 days to measure (**A**) ALP activity and (**B**) cell viability, where * *p* < 0.05, ** *p* < 0.01, *** *p* < 0.001, versus control by one-way ANOVA.

**Figure 7:**
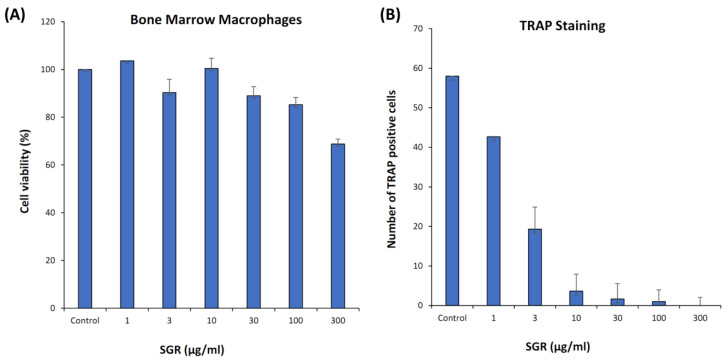
Effects of SGR extract on osteoclast activity. Bone marrow macrophages (BMMs) were treated with SGR extract (1–300 μg/mL) in osteoclast induction medium for 5 days. (**A**) Cell viability and (**B**) number of TRAP-positive cells.

The osteogenic effect of SGR in the pre-osteoblastic MC3T3-E1 cell-line was evaluated. SGR extract enhanced ALP activity significantly without inducing cytotoxic effects (Figure 6A,B). Mouse bone marrow macrophages (BMM) treated with SGR extracts (1–300 μg/mL) were TRAP-stained and osteoclasts were counted after 5 days. SGR-treated cells had showed osteoclast-modulating effects on BMM and at a concentration of 1 μg/mL, no cytotoxicity was seen when compared to the control (Figure 7A,B).

## 3. Discussion 

SGR and its active compounds have been widely used over the last several years as an intervention for many disorders including arthritis treatment. The present study has identified a diverse array of bioactive compounds within SGR, including flavonoids, astilbin, sterols, stilbene, and a sesquiterpene lactone. A total of 14 specific active compounds have been identified based on oral bioavailability (OB ≥ 30%) and drug likeness (DL ≥ 0.18) criteria. Common target genes (59) were obtained from the overlapping of active compounds and disease, indicating that disease genes interact with SGR active compounds. GO enrichment analysis by g-Profiler showed numerous targets were involved in response to oxygen and wound healing in BP, localized to membrane rafts and extracellular regions in CC and associated with cytokine receptors and protein binding in MF. 

Flavonoids identified in SGR have been extensively studied for their anti-inflammatory and antioxidant properties. Compounds such as quercetin, naringenin, astilbin, and taxifolin have demonstrated their ability to inhibit the production of pro-inflammatory cytokines, such as TNF-α, IL-1β, and IL6, which are the key players in OA and RA pathogenesis causing inflammation, cartilage degradation, and bone erosion [10,19,20]. Matrix metalloproteinases (MMPs) degrade the extracellular matrix involved in cartilage destruction in OA, whereas, in RA, these enzymes contribute to joint damage by degradation of the synovial tissue [21,22]. Flavonoids in SGR have been shown to protect articular cartilage by suppressing matrix metalloproteinases (MMPs), enzymes involved in cartilage degradation. SGR active compounds such as quercetin, naringenin, beta-sitosterol, and epicatechin interact with a group of proteins, such as TNF, AKT1, IL6, IL-1β, VEGFA, TP53, CASP3, PTGS2, JUN, and EGF, to regulate/modulate abnormal immune responses observed in arthritis. These targets, such as tumor necrosis factor (TNF), RAC-alpha serine/threonine-protein kinase (AKT1), interleukin-6 (IL6), interleukin-1 beta (IL-1β), vascular endothelial growth factor A (VEGFA), cellular tumor antigen p53 (TP53), caspase-3 (CNASP3), prostaglandin G/H synthase 2 (PTGS2), transcription factor AP-1 (JUN), and pro-epidermal growth factor (EGF), are involved in inflammation, cell survival, growth, and apoptosis. As these compounds influence the arthritis progression, the interaction of four active compounds with nine targets has been investigated by using molecular docking.

Results indicated that quercetin and β-sitosterol exhibited stronger binding energy values as compared to others. The choice of quercetin for docking was based on the previous literature [23,24]. The 24*R* enantiomer of β-sitosterol (PubChem CID: 222284) was selected for docking since it is predominantly found in herbal medicines and has exhibited good biological activity [25]. The enantiomers of (+)-epicatechin (2*S*,3*S*) and naringenin (2*S*) were chosen as they exhibited superior binding affinities and favorable interactions with the protein’s binding pocket, which may contribute to higher potency [26,27]. SGR active compounds can attenuate the severity of arthritis pain by regulating/modulating gene expression levels of TNF, TP53, and JUN. Quercetin (Que), a flavonoid compound with multiple biological activities, can be another potential effector that inhibits the release of pro-inflammatory cytokines (TNF-α, IL-1β, IL6, IL8, IL13, IL17) by activating SIRT1 [28,29]. Que prevents the degradation of the extracellular matrix of cartilage, thereby protecting articular cartilage and delaying the progression of osteoarthritis [30]. Que impacts by blocking the p38 MAPK signal pathway involved in mineralization and differentiation [31]. In vivo, que could reduce the severity of pain by blocking the Akt/NF-B signaling pathway, thus reducing the inflammatory effects of IL-1β and TNF-α on rat chondrocytes [32,33]. By blocking the Caspase-3 pathway, que reduced chondrocyte death by reducing excessive intracellular ROS generation and restored mitochondrial membrane potential. Que has demonstrated immunomodulatory effects on the polarization of synovial macrophages to M2 macrophages, which has helped chondrocytes to generate a pro-chondrogenic milieu and facilitates cartilage repair [34]. Que can be a desired candidate for arthritis treatment through various mechanisms by improving the antioxidant system, regulating immune system reactions, and affecting signaling pathways related to joint and cartilage repair [35]. 

Sterols such as sitosterol, β-sitosterol, and stigmasterol have been reported to inhibit the production of inflammatory mediators and to protect chondrocytes from oxidative stress. β-sitosterol significantly enhances in vitro osteoblastogenic activity by up-regulating the expression of marker genes such as alkaline phosphatase, *runx2*, *osx*, and type1 collagenase [36]. Other bioactive compounds such as dihydroresveratrol and enhydrin have antioxidant, anti-inflammatory, and collagen-protection activities. In osteoarthritic rats, (-)-epicatechin isolated from *Marindo citrifolia* extract can enhance ALP activity to support bone regeneration, integrity, and strength through anti-inflammatory and anti-oxidative pathways [37,38]. Derivatives and isomers of (-)-epicatechin such as (-)-epicatechin-3-O-β-D-allopyranoside and epicatechin gallate have also been reported to enhance bone formation, stimulating osteoblast differentiation through *Runx2* [39]. Naringenin acts as a non-steroid drug that may relieve inflammation and pain by suppressing inflammatory cytokines such as TNF-α and IL-6 [40]. Naringenin enhanced osteoblastogenesis and bone formation through the BMP-2/p38MAPK/Runx2/Osx, SDF-1/CXCR4, and PI3K/Akt/c-Fos/c-Jun/AP-1 signaling pathways. Naringenin also inhibited osteoclastogenesis and bone resorption by inhibiting inflammation and the RANKL pathway [41]. 

AKT1 (RAC-α serine/threonine-protein kinase), a unique signaling intermediate in osteoblasts, can regulate the differentiation of both osteoblasts and osteoclasts and can also activate specific downstream targets by interacting with NF-κB, mTOR, and TP53 pathways. In synovial fluid and osteoarthritis (OA), IL-6 and TNF-α are the key immune mediators and inflammatory markers [42,43,44]. TNF receptors (TNFR1 and TNFR2) in signaling pathways are capable of activating MAPKs and activating the NF-κB pathway through different adaptor proteins. Depending on the cell type and receptor, TNFR signaling may induce apoptotic/survival signals that are proliferative in osteoclasts and/or inhibitory in osteoblasts and osteocytes. TNF-α promotes bone resorption from multiple directions and contributes to progression by directly inducing osteoclast formation via TNFR1 but not TNFR2 and indirectly induces osteoclast formation by TNF-α-induced RANKL expression in osteoblasts and osteocytes [45]. TNF-α and IL-1β, induced by NF-κB, also activates the NF-κB signaling pathway in both OA and RA [46]. In mice, KAG-308 (EP4 selective agonist) administration reduces chondrocyte hypertrophy and TNF secretion, leading to the suppression of knee osteoarthritis [47]. IL-6 not only affects the immune system, but also biological and physiological systems in cells regulating growth, gene activation, proliferation, survival, and differentiation. Thus, TNF-α can induce the production of IL-6 and activate the protease that decomposes cartilage and synovium.

Interleukin-1 beta (IL-1β) induces prostaglandin (PTGS2) synthesis, which has both catabolic and anabolic effects in cartilage [42,48]. As bone repair depends largely on the VEGF signaling pathway, it has been investigated in tibia cortical bone defect models; blocking extracellular VEGF by soluble fms-like tyrosine kinase-1 (sFlt1) has decreased bone regeneration, whereas the administration of VEGF has increased bone mineralization [49]. TP53 may reduce inflammation by working synergistically with TLR5 by increasing the expression of > 2000 genes, mostly related to immunity/inflammation [50,51]. In addition, both TP53 and cyclin D1 have a role in the progression of arthritis by regulating cell apoptosis [52]. EGF affects processes that are associated with bone healing in the case of surgically induced osteonecrosis of the femoral head (ONFH) by osteoblast differentiation and bone resorption [53]. 

The Activator Protein-1 (AP-1) transcription factor (TF) family, composed of a variety of members including c-JUN, c-FOS, and ATF, is involved in mediating many biological processes such as proliferation, differentiation, and cell death [54]. The Jun and Fos family are basic leucine zipper proteins that play specific roles in bone remodeling, as demonstrated by several loss- or gain-of-function studies in mice [55]. It has been demonstrated that mice lacking *JunB* showed osteopenia due to cell-autonomous defects of osteoblasts and osteoclasts [56]. Caspase-3 is a critical enzyme for apoptosis/cell survival and maintaining both bone development and metabolism. Casp3−/− mice exhibited significant bone defects during early development, whereas Casp3+/− mice displayed a decreased bone mineral density (BMD) with age, a defect consistent with osteoporosis. Caspase-3 activity is required for the functional differentiation of bone marrow-derived mesenchymal stem cells (BMSCs). Increased replicative senescence and the altered expression of Runx2/Cbfa1 through upregulated TGF-β/Smad2 signaling may contribute to the impaired osteogenic differentiation of BMSCs [57]. These results specify that targets are closely related to the TNF, NF-kB, PI3K-Akt, VEGF, and AGE-RAGE signaling pathways, and lipids and atherosclerosis are primarily involved. Binding affinity predictions of selected docked compounds revealed that interaction is stronger and can be validated by experimental studies. In vitro analysis showed that alkaline phosphatase (ALP) activity has increased in SGR-treated samples, which are the indicator of osteoblast differentiation. It suppresses osteoclastogenesis, as indicated in TRAP staining on bone marrow macrophages. While ALP and TRAP are primarily associated with bone metabolism, they can indirectly influence biological processes through their involvement in signaling pathways and their potential roles in various diseases. Elevated levels of ALP and TRAP have been associated with inflammatory conditions, which can involve the upregulation of cytokines such as TNF, IL6, and IL-1β [58]. These enzymes can potentially affect the expression of genes like *AKT1*, *TP53*, and *CASP3* by influencing cell survival and apoptosis pathways [59]. ALP and TRAP may play roles in vascular biology, potentially altering the expression of VEGF [60]. These findings suggest that SGR treatment for arthritis patients may have a positive impact. Taken together, SGR might show therapeutic efficacy through blocking the TFN, NF-kB, and MAPK inflammatory pathways to regulate the expression or activities of the immune system.

## 4. Material and Methods

### 4.1. Experimental Procedures

**Figure 8:**
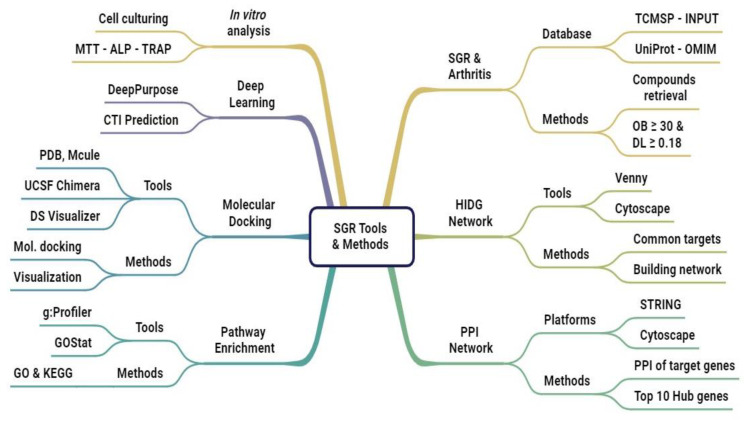
Flowchart of this study.

The workflow of the research study is presented in Figure 8. The methodology comprises data compilation, data preprocessing, network analysis, molecular docking (MD), and deep learning (DL).

#### 4.1.1. Data Acquisition of SGR

Active compounds and structures of the traditional medicinal herb, SGR, were retrieved from five different in silico open sources: (1) Traditional Chinese Medicine Systems Pharmacology (TCMSP) “URL tcmsp-e.com (accessed on 6 November 2023)”; (2) Traditional Chinese Medicine Information Database (TCM-ID) “URL https://bidd.group/TCMID/ (accessed on 6 November 2023); (3) HERB “URL http://herb.ac.cn/ (accessed on 6 November 2023)”; (4) HIT-index “URL http://www.badd-cao.net:2345/ (accessed on 6 November 2023)”; and (5) INPUT “URL https://cbcb.cdutcm.edu.cn/INPUT/Home/ (accessed on 6 November 2023)”. All the platforms were freely available, specialized and curated for providing active herb compounds as well as target information based on evidence from the literature. Oral bioavailability (OB ≥ 30) and drug-likeness (DL ≥ 0.18) were set as the thresholds for the screening of active compounds. Compounds that did not pass the thresholds were excluded from the criteria.

#### 4.1.2. Potential Targets of Arthritis

Under the keywords “osteoarthritis”, three comprehensive, reliable, and user-friendly resources—(1) The Human Gene Database, GeneCards “URL https://www.genecards.org/ (accessed on 9 December 2023)”; (2) the Online Mendelian Inheritance in Man (OMIM) database “URL http://www.omim.org (accessed on 9 December 2023)”; and (3) the Therapeutic Target database “URL https://db.idrblab.net/ttd/ (accessed on 7 January 2024)”, were used to evaluate the data. For normalization and removing any errors, all the targets were checked using UniProt “URL http://www.uniprot.org/ (accessed on 7 January 2024)”. 

#### 4.1.3. Common Target Determination and Network Construction 

The common targets between the SBG active compounds and arthritis were recognized by Venny 2.1 “URL https://bioinfogp.cnb.csic.es/tools/venny/ (accessed on 10 February 2024)”. Data imported to Cytoscape 3.10.0 software “URL http://cytoscape.org/ (accessed on 10 February 2024)” were used to build and visualize the herb–ingredients–gene–disease network. 

#### 4.1.4. Network Visualization and PPI Analysis

The protein–protein interaction (PPI) of target genes was mapped by the STRING platform “URL https://string-db.org/ (accessed on 20 March 2024)” to determine their relationships and interactions. The cytoHubba plugin was used for the identification of the top connected hub genes/biomarkers. 

#### 4.1.5. Functional Annotation of Common Target Genes

Gene Ontology (GO) and Kyoto Encyclopedia of Genes and Genomes (KEGG) pathway analysis of common targets was carried out by g-profiler “URL https://biit.cs.ut.ee/gprofiler/gost (accessed on 20 March 2024)”. GOStat was used as a useful tool to find biological processes of a group of target genes. The species was set to “Homo sapiens”, with *p* = 0.05 selected.

#### 4.1.6. Molecular Docking

Molecular docking was used for determining the associations between the active compounds and core hub targets. The crystal structure of the 9 hub targets were obtained from the Protein Data Bank (PDB) “URL https://www.rcsb.org/ (accessed on 12 April 2024)”. Structures were prepared for molecular docking with UCSF chimera “URL https://www.cgl.ucsf.edu/chimera/ (accessed on 12 April 2024)”. Prior to molecular docking, the receptor and ligand structures were prepared. The receptor preparation involved removing the ligand, adding missing hydrogens, and resolving any structural inconsistencies. The “DockPrep” tool was utilized for these tasks, ensuring the receptor was ready for docking simulations. For the ligand, hydrogens were added and charges were calculated using the “Add Charge” tool within Chimera. This tool assigned appropriate atomic types, bond types, and charges, ensuring accurate representations of the ligand’s electrostatic properties. The XYZ coordinates for each protein are provided in Supplementary Table S6. An integrated computer-aided molecular platform, Mcule “URL https://mcule.com/ (accessed on 25 April 2024)”, was used for molecular docking and the results were analyzed and visualized by BIOVIA Discovery Studio. 

#### 4.1.7. Compound Targets Predictions Based on MPNN and CNN

**Figure 9:**
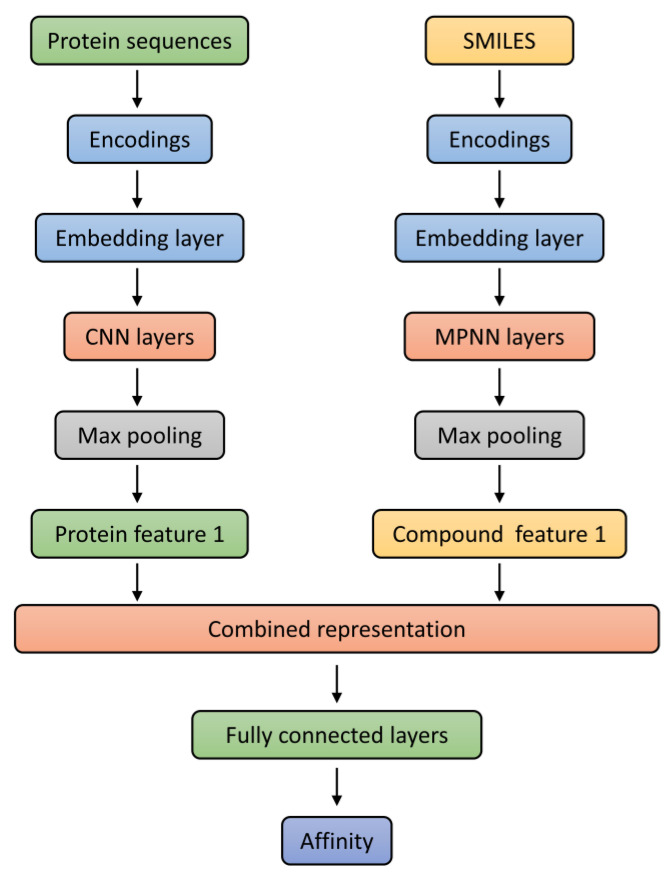
Architecture of deep neural network for compound target prediction.

DeepPurpose, “URL https://github.com/kexinhuang12345/DeepPurpose (accessed on 27 April 2024)”, a deep learning-based molecular model, was designed to predict drug–target interaction (DTI) binding affinities. Multi-model approaches are used for processing SMILES and protein sequences separately before combining them for a final prediction. Pre-trained benchmark Davis and KIBA models were used, which consist of datasets with dissociation constant (Kd) and bioactivity types (IC_50_, Ki, and Kd), respectively, to facilitate drug discovery procedures. The sequence information of compounds (SMILES) and targets (protein sequences) were converted into embeddings and separately fed into a type of graph neural network (GNN) called a message passing neural network (MPNN) and convolution neural network (CNN) blocks, which aim to learn representations from SMILES strings and protein sequences. A max pooling layer was added to generate compact compounds and protein feature representations. Both compounds and a protein feature representation combined into a single vector were fed into a fully connected (FC) layer to obtain high-level features for predicting interaction scores of compounds with target proteins (Figure 9). 

#### 4.1.8. Plant Extract Preparation

The dried root of *Smilax glabra* used in the experiment was provided by Yaksudang Pharmaceutical Co., Ltd. (Seoul, Republic of Korea). The biological identity of the plant material was authenticated by Prof. Donghun Lee, Dept. of Herbal Pharmacology, College of Korean Medicine, Gachon University, and the voucher specimen number was deposited (No. D210609002). The SGB roots were ground to a fine powder, and extracted with 30% EtOH in a water bath at 100 °C for three hours under reflux conditions using a solid-to-solvent ratio of 1:10. The resulting extract was filtered, concentrated under reduced pressure, and lyophilized at −80 °C. The final yield of SGB was 11.23%.

#### 4.1.9. Cell Culturing and ALP Activity

MC3T3-E1 cells (subclone 4, #CRL-2593) were obtained from biological resource center, American Type Culture Collection (ATCC^®^, Manassas, VA, USA). MC3T3-E1 cells (1 × 10^4^/well) in a 96-well plate were cultured in an αMEM medium (10% FBS, 100 U/mL penicillin and 100 μg/mL streptomycin) at 37 °C, 5% CO_2_, for 24 h. Osteogenic differentiation was induced by culturing cells in medium supplemented with ascorbic acid (50 μg/mL) and β-glycerolphosphate (10 mM) and treated with different concentrations of SGR extract (1, 3, 10, 30, 100, and 300 μg/mL) for 4 days. Cells were rinsed with phosphate-buffered saline, lysed with RIPA buffer; p-Nitrophenyl Phosphate (pNPP; 100 μL) (Thermo Scientific, Waltham, MA, USA, 37621) was added and incubated for 1 h. ALP activity in the cells was measured at 405 nm by using an Epoch2 microplate reader (BioTek, Winooski, VT, USA).

#### 4.1.10. Cell Viability Assay

For cell viability, harvested cells (1 × 10^4^/well) were treated with different concentrations of SGR extract (1–300 μg/mL) for 5 days. Cell viability was assessed by EZ-cytox assay and optical density was measured at 450 nm. Untreated cells were used as the control.

#### 4.1.11. TRAP Staining

Bone marrow macrophages (BMM) were collected from 5-week-old mice and spun at 1300 rpm for 8 min at room temperature, supplemented with M-CSF (20 ng/mL) and RANKL (100 ng/mL), and treated with different concentrations of SGR extract (1–300 μg/mL) for 5 days. TRAP staining of osteoclasts was carried out by using a kit (Takara, Seoul, Republic of Korea, MK300) and the TRAP-positive cell number was counted and compared with control.

## 5. Conclusions

The identification of bioactive compounds in SGR provides a strong foundation for the development of novel therapeutics for arthritis. By targeting multiple pathways involved in pathogenesis, SGR may offer a synergistic approach for disease management and treatment. Preclinical studies evaluating the efficacy and safety of SGR compounds in animal models are necessary. Additionally, human clinical trials are essential to assess the long-term benefits and potential side effects of SGR-based therapies. Active research is needed to identify potential key markers to differentiate between OA and RA and to uncover therapeutic targets that selectively modulate disease-specific pathways. Developing combination therapies by using SGR compounds can address both inflammatory and degenerative aspects of arthritis. 

## Figures and Tables

**Table 1:** Active compounds of SGR.

Pubchem ID	Names	Structure	Category	Canonical Smiles	Oral Bioavailability OB (%)	Drug Likeness (DL)
442437	Neoastilbin	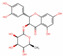	Flavonoid	CC1C(C(C(C(O1)OC2C(OC3=CC(=CC(=C3C2=O)O)O)C4=CC(=C(C=C4)O)O)O)O)O	40.54	0.74
129394	4,7-dihydroxy-5-methoxyl-6-methyl-8-formyl-flavan	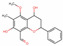	Flavonoid	CC1=C(C(=C2C(=C1OC)C(CC(O2)C3=CC=CC=C3)O)C=O)O	37.03	0.28
12303645	Sitosterol	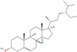	Sterol	CCC(CCC(C)C1CCC2C1(CCC3C2CC=C4C3(CCC(C4)O)C)C)C(C)C	36.91	0.75
222284	Beta-sitosterol	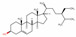	Sterol	CCC(CCC(C)C1CCC2C1(CCC3C2CC=C4C3(CCC(C4)O)C)C)C(C)C	36.91	0.75
119258	Astilbin	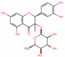	Flavonoid	CC1C(C(C(C(O1)OC2C(OC3=CC(=CC(=C3C2=O)O)O)C4=CC(=C(C=C4)O)O)O)O)O	36.46	0.74
101937309	Isoengelitin	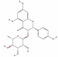	Flavonoid	CC1C(C(C(C(O1)OC2C(OC3=CC(=CC(=C3C2=O)O)O)C4=CC=C(C=C4)O)O)O)O	34.65	0.7
182232	(+)-Epicatechin	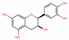	Flavonoid	C1C(C(OC2=CC(=CC(=C21)O)O)C3=CC(=C(C=C3)O)O)O	48.96	0.24
185914	Dihydroresveratrol	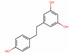	Stilbene	C1=CC(=CC=C1CCC2=CC(=CC(=C2)O)O)O	87.27	0.29
99474	Diosgenin	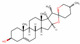	Flavonoid	CC1CCC2(C(C3C(O2)CC4C3(CCC5C4CC=C6C5(CCC(C6)O)C)C)C)OC1	80.88	0.81
443758	Cis-dihydroquercetin	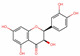	Flavonoid	C1=CC(=C(C=C1C2C(C(=O)C3=C(C=C(C=C3O2)O)O)O)O)O	66.44	0.27
5320468	(2*R*,3*R*)-2-(3,5-dihydroxyphenyl)-3,5,7-trihydroxychroman-4-one	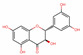	Flavonoid	C1=C(C=C(C=C1O)O)C2C(C(=O)C3=C(C=C(C=C3O2)O)O)O	63.17	0.27
712316	(-)-Taxifolin	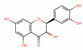	Flavonoid	C1=CC(=C(C=C1C2C(C(=O)C3=C(C=C(C=C3O2)O)O)O)O)O	60.51	0.27
439246	Naringenin	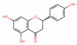	Flavonoid	C1C(OC2=CC(=CC(=C2C1=O)O)O)C3=CC=C(C=C3)O	59.29	0.21
439533	Taxifolin	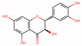	Flavonoid	C1=CC(=C(C=C1C2C(C(=O)C3=C(C=C(C=C3O2)O)O)O)O)O	57.84	0.27
5280343	Quercetin	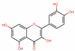	Flavonoid	C1=CC(=C(C=C1C2=C(C(=O)C3=C(C=C(C=C3O2)O)O)O)O)O	46.43	0.28
5280794	Stigmasterol	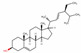	Sterol	CCC(C=CC(C)C1CCC2C1(CCC3C2CC=C4C3(CCC(C4)O)C)C)C(C)C	43.83	0.76
5377450	Enhydrin	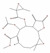	Lactone	CC1C(O1)(C)C(=O)OC2C3C(C4C(O4)(CCC=C(C2OC(=O)C)C(=O)OC)C)OC(=O)C3=C	40.56	0.74

**Table 2:** A heat map of the four active components of SGR and key targets. The size of the binding energy (kcal/mol) is shown with the absolute value; the redder shade specifies an increasing stability of the combination of the component and the target protein. A color change from green to red indicates the binding energy changing from high to low.

PDB ID	Target	Quercetin	Naringenin	β-Sitosterol	Epicatechin
2AZ5	TNF	−7.8	−7.1	−8.6	−7.8
1UNQ	AKT1	−5.7	−5.4	−5.4	−5.7
1IL6	IL6	−7.2	−6.9	−4.8	−6.8
6Y8M	IL-1β	−6.5	−5.7	−5.5	−6.6
3QTK	VEGFA	−4.9	−4.8	−5.5	−4.6
3DCY	TP53	−8.0	−7.6	−7.4	−7.6
1NMS	CASP3	−6.7	−6.1	−6.9	−6.5
5KIR	PTGS2	−8.2	−5.5	−5.5	−8.7
1PMN	JUN	−8.5	−7.2	−8.9	−7.8

**Table 3:** Compound target interaction prediction of active compounds of SGR with Davis (pKd in nM) and KIBA scores. Binding affinity is measured by equilibrium dissociation constant (Kd).

Targets	Binding Affinity by Davis Model				Binding Affinity by KIBA Model			
	Quercetin	Naringenin	β-Sitosterol	Epicatechin	Quercetin	Naringenin	β-Sitosterol	Epicatechin
TNF	6.055	5.989	6.631	6.063	11.186	11.228	11.349	11.182
AKT1	5.997	5.467	6.573	6.005	11.064	11.120	11.227	11.060
IL6	5.569	5.958	6.601	5.514	11.205	11.247	11.368	11.201
IL-1β	5.650	5.965	6.608	6.040	11.218	11.260	11.381	11.214
VEGFA	6.131	6.064	6.706	6.138	11.378	11.419	11.540	11.374
TP53	6.134	6.067	6.709	6.141	11.307	11.349	11.470	11.303
CASP3	6.057	5.990	5.929	6.065	11.170	11.212	11.327	11.166
PTGS2	6.068	6.001	6.644	6.076	11.342	11.383	11.504	11.338
JUN	5.697	6.017	5.712	5.642	11.265	11.306	11.428	11.261

## Data Availability

The Davis and KIBA datasets can be found at “URL https://github.com/SI319 (accessed on 27 April 2024)”.

## Supplementary Materials

The following supporting information can be downloaded at https://www.mdpi.com/article/10.3390/ph17101285/s1. Table S1: Pharmacological and molecular property profiling of SGB compounds; Table S2: Common Targets of SGB and Arthritis; Table S3: Frequency of compounds and Targets; Table S4: Biological interactions showing various attributes by String Database; Table S5: Compound target interaction prediction of active compounds of SGB; Table S6: The Cartesian coordinates (XYZ) of proteins determined by Grid box method; Figure S1: Interaction network of the active compounds of SGB with common targets; Figure S2: Length distribution of SMILES and protein sequences.
