# Supplementary material for: Mechanistic Exploration of Smilax glabra Roxb. in Osteoarthritis: Insights from Network Pharmacology, Molecular Docking, and In Vitro Validation
Source: Pharmaceuticals (Basel). 2024 Sep 27;17(10):1285. doi: 10.3390/ph17101285 (PMC11510151; doi:10.3390/ph17101285)
Supplement: Supplementary file 1 [file pharmaceuticals-17-01285-s001.zip › Supplementary SGR Figures.pdf]

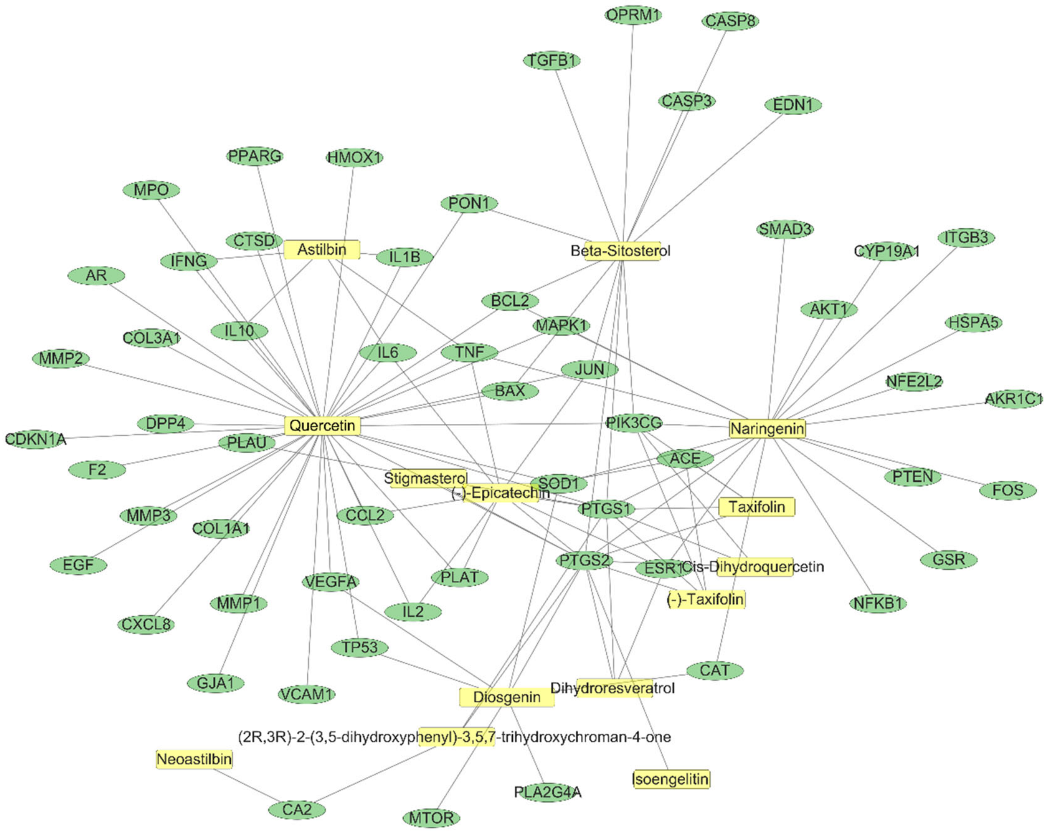

Supplementary Figure S1. Interaction network of the active compounds of SGB with common targets.

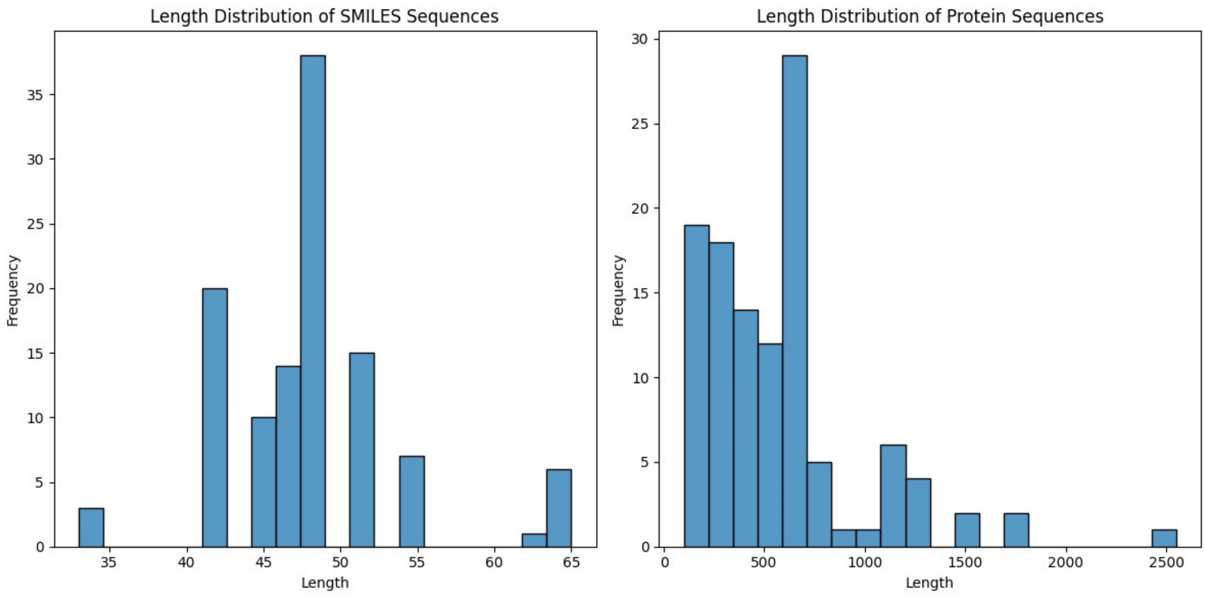

Supplementary Figure S2. Length distribution of SMILES and protein sequences.
